# How Light at Night Sets the Circalunar Clock in the Marine Midge *Clunio marinus*

**DOI:** 10.1177/07487304241286936

**Published:** 2024-11-06

**Authors:** Carolina M. Peralta, Eric Feunteun, Julien Guillaudeau, Dušica Briševac, Tobias S. Kaiser

**Affiliations:** * Max Planck Research Group Biological Clocks, Max Planck Institute for Evolutionary Biology, Plön, Germany; † UMR Biologie des Organismes et Ecosystèmes Aquatiques, (MNHN, CNRS, SU, IRD, UCN, UA), Dinard, France; ‡ Centre de Géoécologie Littorale (EPHE-PSL), Dinard, France; § Muséum National d’Histoire Naturelle, Dinard, France

**Keywords:** circasemilunar clock, zeitgeber, entrainment, moonlight, light-intensity modulation by tides, adaptive phase response, coincidence detection, circadian gating, sensory ecology

## Abstract

Many organisms inhabiting the interface between land and sea have evolved biological clocks corresponding to the period of the semilunar (14.77 days) or the lunar (29.53 days) cycle. Since tidal amplitude is modulated across the lunar cycle, these circasemilunar or circalunar clocks not only allow organisms to adapt to the lunar cycle, but also to specific tidal situations. Biological clocks are synchronized to external cycles via environmental cues called *zeitgebers*. Here, we explore how light at night sets the circalunar and circasemilunar clocks of *Clunio marinus*, a marine insect that relies on these clocks to control timing of emergence. We first characterized how moonlight intensity is modulated by the tides by measuring light intensity in the natural habitat of *C. marinus*. In laboratory experiments, we then explored how different moonlight treatments set the phase of the clocks of two *C. marinus* strains, one with a lunar rhythm and one with a semilunar rhythm. Light intensity alone does not affect the phase of the lunar rhythm. Presenting moonlight during different 2-h or 4-h windows during the night shows that (1) the required duration of moonlight is strain-specific, (2) there are strain-specific moonlight sensitivity windows and (3) timing of moonlight can shift the phase of the lunar rhythm to stay synchronized with the lowest low tides. Experiments simulating natural moonlight patterns confirm that the phase is set by the timing of moonlight. Simulating natural moonlight at field-observed intensities leads to the best synchronization. Taken together, we show that there is a complex and strain-specific integration of intensity, duration and timing of light at night to precisely entrain the lunar and semilunar rhythms. The observed fine-tuning of the rhythms under natural moonlight regimes lays the foundation for a better chronobiological and genetic dissection of the circa(semi)lunar clock in *C. marinus*.

The celestial movements of the earth and the moon entail regular changes in the environment, such as night and day, the seasons, the lunar cycle and the tidal cycle. Organisms have evolved corresponding biological clocks, that is, endogenous time-keeping mechanisms, which are highly adaptive for each particular habitat, as they permit species to anticipate regular changes and time behavior or physiology accordingly. Moreover, they allow populations of individuals to synchronize important life history events such as reproduction. For each of the above-mentioned geophysical cycles, there is a corresponding biological clock. The circadian clock (24 h) is the most widespread across the animal kingdom and by far the best-studied (Dunlap, 1999; Kaiser and Neumann, 2021). However, many organisms inhabiting the interface between land and sea have evolved biological clocks that allow them to adapt to the tides. These include the circatidal clock (12.4 h), the circasemilunar clock (14.77 days) and the circalunar clock (29.53 days) (reviewed in Neumann, 2014). Circasemilunar and circalunar clocks help organisms to adapt to the tides, because the tidal amplitude is modulated across the lunar cycle, being highest during the spring tide days just after full moon and new moon.

In this study we focus on the circasemilunar and circalunar clock of the marine insect *Clunio marinus. C. marinus* larvae and pupae settle in the lower intertidal, where they are almost constantly submerged (Neumann, 2014). However, the adults need the larval habitat to be exposed in order for oviposition to be successful. Exposure of the larval habitat occurs reliably every month during the spring tides, when tidal amplitude is highest. In line with that, *C. marinus* has a short adult lifespan of few hours, which is only used for reproduction and which is timed exactly to spring tide low tides. This is achieved via a combination of circasemilunar or circalunar and circadian clocks. The circalunar or circasemilunar clock synchronizes development and maturation with the spring tide days. Notably, there are *C. marinus* strains with a circalunar clock which only emerge during full moon or new moon, and there are strains with a circasemilunar clock which emerge during both full and new moon (Kaiser et al., 2021). The circadian clock gates adult emergence to one of the two daily low tides.

Biological clocks are synchronized to external environmental cycles via reliable timing cues, called *zeitgebers*. Once the zeitgeber effectively entrains (synchronizes) the clock, the endogenous rhythm will have a stable phase-relationship to the zeitgeber, the so-called phase of entrainment (Aschoff and Pohl, 1978). Thus, the zeitgeber is crucial for the synchronization of physiological processes to the external environmental cycles as it conveys information about the phase to the biological clock (Naylor, 2010).

The relevance of moonlight as an effective zeitgeber for lunar rhythms was first described in 1960 in the annelid *Platynereis* (Hauenschild, 1960). Since then, moonlight as a zeitgeber has been validated for many other species such as the brown algae *Dictyota dichotoma* (Bünning and Müller, 1961), the crab *Sesarma haematocheir* (Saigusa, 1980) and different *Clunio* species (*C. marinus*, *C. mediterraneus* and *C. tsushimensis*, reviewed in Neumann, 2014). While in this study we focus on entrainment by moonlight, it is noteworthy that the zeitgebers for setting *Clunio*’s circalunar clock also include tidal cycles of mechanical agitation (Neumann, 1978) and tidal temperature cycles (Neumann and Heimbach, 1979; reviewed in Kaiser and Neumann, 2021).

Previous studies in *Clunio* started to explore how artificial moonlight—or more generally light at night—entrains the circalunar clock. Importantly, in this context “light at night” does not refer to “artificial light at night” (ALAN) from the field of light pollution, but to the fact that light was experimentally administered during the night, in various intensities and patterns that do not necessarily correspond to natural moonlight. It was shown that one night of continuous artificial moonlight is insufficient to properly synchronize a semilunar strain of *C. marinus* (Neumann, 1976). This led to the suggestion that light perception might be combined with some sort of counting mechanism (Neumann, 1987). Three, four or six nights with artificial moonlight are sufficient to entrain the circasemilunar or circalunar clocks (Neumann, 1966). Another major result was that a circadian clock regulates the perception of light at night (Neumann, 1989). *C. tsushimensis* was exposed to four consecutive days of constant darkness (DD) and artificial moonlight at different times of the subjective day and night. This has shown that only light given in the subjective night effectively entrains the circalunar clock (Neumann, 1995). The fact that this regulation of sensitivity works under DD suggests that it is controlled by a circadian clock. Further studies on which particular times at night set the phase were performed by providing windows of light at night in shorter periods over the night. Exposing *C. tsushimensis* to a light dark-cycle of 12:12 and 3 h of light at night shows that only moonlight in the middle of the night works as an effective zeitgeber (Neumann, 1987). Exposing the semilunar Santander (San) strain of *C. marinus* to 4 h of light at night, suggests that moonlight is only perceived in the second half of the night (Neumann, 1969).

The phase relationship between artificial moonlight and emergence peak can be modified by changing different components of the artificial moonlight such as the number of successive nights with light at night (Neumann, 1976), varying the period of the light-dark cycle (Neumann, 1989; Neumann and Heimbach, 1979) and by a more complex simulated moonlight program (Neumann, 1985). The latter includes the daily shift in the rise and fall of the moon, as well as three different levels of light intensity corresponding to different lunar phases. When the tides were mimicked by restricting light administration to the early, middle or late hours of the complex simulated moonlight program, the adult emergence peak did not follow the brightest moonlight, but shifted with the timing of light at night (Neumann, 1985). Finally, laboratory strains of *C. marinus* from different geographical locations show a strain-specific phase relationship between the artificial moonlight stimulus and the emergence peak in the laboratory (Neumann, 1966). It was shown that there is a good correlation between the time from the light stimulus to the emergence peaks in the laboratory and the time from midnight low-tides (when moonlight is presumably best perceived) to emergence in the field (Kaiser et al., 2011). Thus, *C. marinus* populations from different geographic origins show local adaptation in the phase relationship between moonlight perception and adult emergence.

Open questions remain, such as if and how light is modulated by the tides and to what extent the windows of light sensitivity vary across populations of *C. marinus*. It is also unclear which components of the moonlight—intensity, duration or daily timing—set the phase of emergence. In this study, we show by field measurements how moonlight intensity reaching the intertidal is modulated by the tides. In corresponding laboratory experiments we explored how different moonlight treatments set the phase of the circa(semi)lunar clock. We assessed two laboratory strains which have a lunar or a semilunar rhythm. We show that there is a complex and strain-specific interaction of intensity, duration and timing of light at night in setting the strength and phase of entrainment.

## Methods

### Light in the Intertidal Zone

In order to evaluate moonlight modulation by the tides in a *C. marinus* habitat, a RAMSES-ACC-VIS hyperspectral radiometer (TriOS GmbH, Germany) was placed at Rocher de Bizeux in Dinard (see coordinates in Table S1, Figure S1). The sensor was attached to the rock at 1 m below the low water of the theoretical maximal tidal range, so it was permanently submerged. The site is near St. Briac-sur-Mer, from which a *C. marinus* strain has been obtained and described (Bria-1SL, Kaiser et al., 2021) . The radiometer measured 192 wavelengths between 317 and 953 nm, at a resolution of ~3 nm (data published on Max Planck Repository Edmond, https://doi.org/10.17617/3.6OXUES). Light was recorded every 5 min, over nearly four consecutive lunar months (106 nights) from 17 October 2013 to 1 February 2014. All timestamps were recorded in the time zone in which the device was deployed, which was in Central European Summer Time (CEST) on 17 October 2013, and later converted to Coordinated Universal Time (UTC). A plot of the raw data is given in Figure S2a and the averaged daylight and moonlight spectra in Figures S2b and S2c.

To investigate moonlight properties, we looked at changes in light intensity during the night over several months. As light intensity was low (i.e., for 502 nm on a full moon night, intensity was between 9.99 × 10^−6^ and 0.001 mW/m^−2^/nm^−1^), raw values were averaged in 30-min intervals and values at each measured wavelength were summed in 100 nm bins. The highest light intensity detected in the middle of the night was of 0.06 mW/m^−2^/nm^−1^, on full moon (night between 17 November 2013 and 18 November 2013). Values above this threshold were considered as daylight and used to empirically define the limits between night (Figure S3a-b) and day (Figure S3c-d). These limits happened to fall at 0600 and 1800, based on the averaged 30-min intervals.

The strongest intensity and clearest moonlight signal were observed between November 2013 and January 2014 (Figure S3a-b). Therefore, we subsetted the data for this time-frame (from new moon 3 November 2013 to new moon 2 January 2014, Figure 1a). In further text we referred to this data set as “Dinard-underwater.”

**Figure 1.**
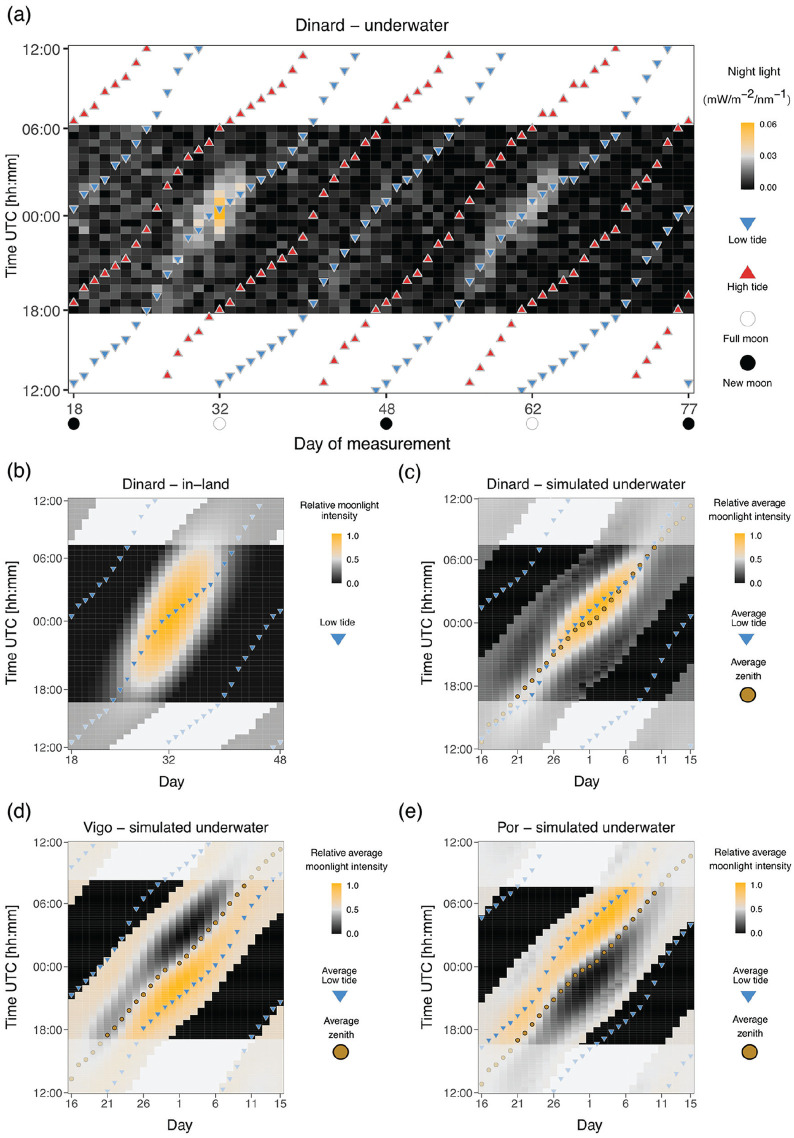
Moonlight intensity in the intertidal zone is modulated by the tides. **(a)** Heatmap of light intensity detected at night with a submerged radiometer in the intertidal region of Dinard. Days of two consecutive lunar months from new moon to new moon (3 November 2013 and 2 January 2014) are shown on the x-axis and time of day on the y axis. Timing of low and high tides is shown as blue and red triangles, respectively. The maximum daily moonlight duration detected was of 4 h 30 min in days close to full moon. **(b)** Heatmap of relative moon intensity in-land for Dinard obtained from the R package *“suncalc.*” One month (from 3 November 2013 to 4 December 2013) is plotted and low tide is shown in blue inverted triangles. Night-time was defined as the time between sunset and sunrise, the times of which were also retrieved from the *“suncalc”* package and averaged for the given month. Moonlight duration in the absence of tides is over 14 h on full moon days. **(c-e)**. Heatmaps of relative average moonlight intensity for three geographic sites, simulated by multiplying normalized water levels and relative moonlight intensity (see Methods). Timing of low tides is shown in blue inverted triangles, moon zenith is shown in a golden circle. **(c)** Dinard (France), **(d)** Vigo (Spain) and **(e)** Port-en-Bessin (France). Due to the effect of the water levels, the timing of moonlight visibility during the night varies greatly across geographical locations.

To assess the relative changes in light intensity over the day per wavelength, we used the raw 5-min light-intensity data set of 106 days (Figure S4). Timing and water level of high and low tides were obtained from www.maree.info (Figure S4) from the closest marine station available, located in St. Malo. The tidal timestamps were rounded to the nearest 5-min interval to match the moonlight data.

### Tidal Modulation of Moonlight

In order to investigate how tides affect the visibility of moonlight in the intertidal region, we inferred the impact of the water column (Copernicus Program ERA5, see details below) on moonlight in-land (“*suncalc*” package, see below), and compared it with the quantified moonlight intensity underwater as measured by the radiometer (“Dinard-underwater” data set).

#### Comparing Underwater and in-Land Moonlight Patterns

We extracted water levels for Dinard (Table S1, Figure S1) from Copernicus Program ERA5 (Hersbach et al., 2020) for the same period of time as the “Dinard-underwater” data set (Figure 1a). The ERA5 data set comprises a reanalysis of water levels (a combination of model data with observations from across the world into a data set using the laws of physics) with a temporal resolution of 10 min in UTC. Low tide and high tide were defined as the maximum and minimum water level in each tidal cycle, respectively (Figure 1a). In order to superimpose the 10 min water level data set to the averaged 30-min light “Dinard-underwater” data set, we assigned the tide timestamp to the closest 30-min interval (Figure 1a).

We also plotted moonlight intensity in-land, that is, without tides (R package *“suncalc*,” Thieurmel and Elmarhraoui, 2022), at the same location over a single lunar cycle from new moon (3 November 2013) to new moon (4 December 2013) (Figure 1b). In further text we referred to this data set as “Dinard in-land.” Relative moonlight intensity was calculated as the fraction of moon illumination in 10 min intervals multiplied by moon altitude, and then normalized to 1. Moon altitude is important here because lower moon altitudes generally result in lower light intensities per surface area, and particularly in the intertidal zone where moonlight may additionally be reflected on the water surface. Low tides (blue triangles) were superimposed as above. In order to determine night-time, sunset and sunrise times were also extracted from the *“suncalc”* package and averaged for the respective time period (3 November 2013 to 4 December 2013).

#### Inferring Light Visibility in the Intertidal of Different Geographical Locations

The timing of the low tides varies across the Atlantic coast, and therefore tidal modulation of moonlight can be expected to differ in different geographic locations. As deploying the radiometer in different geographical locations was not feasible, we explored publicly available water and light data sets to simulate tidal modulation of moonlight. The comparison of field measurements from the intertidal zone (“Dinard-underwater,” Figure 1a) with general moonlight availability (“Dinard-in land,” Figure 1b) suggested that the tides considerably reduce the availability of moonlight. The simulations were set up in a way to reproduce this observed effect (compare Figure 1a to 1c).

We extracted water levels (ERA5, Hersbach et al., 2020) and in-land moonlight intensities (R package *“suncalc*,” Thieurmel and Elmarhraoui, 2022) in 10-min increments over eight lunar cycles from March to October of 2017, a time when *C. marinus* is not expected to be in diapause (Neumann, 1986).

In order to estimate how the water column affects moonlight intensity, we multiplied relative moonlight intensity with water levels scaled to a -1 to 1 range, referred as “simulated underwater” data sets. We then subsetted the data into eight cycles, where each cycle starts at midnight of a full moon day (full moon dates were taken from https://www.timeanddate.com). The eight cycles were averaged into one 30-day cycle (Figure 1c to 1e). Furthermore, we extracted the timing of the low tide as minimal water level value for each tidal cycle, subsetted the data into eight lunar cycles (Day 1 = full moon), and averaged it (Figure 1c to 1e, blue triangles). Finally, we extracted the timing of the highest moonlight intensity (moon zenith) over the eight cycles, and averaged it accordingly (Figure 1c to 1e, golden circles). The analysis was performed for Dinard (“Dinard—simulated underwater,” Figure 1c), Vigo (“Vigo—simulated underwater,” Figure 1d) and Port-en-Bessin (“Por—simulated underwater,” Figure 1e). As the simulated underwater moonlight data represents averages over eight lunar cycles throughout the year, there is no single corresponding daylength. We therefore chose to set the night duration to the same length as in the “Dinard in-land” data set, which allows for comparison. Notably, the middle of the night varies between locations because they are on different geographical longitudes, and we have accounted for this effect.

### *Clunio marinus* Laboratory Culture

Two *C. marinus* laboratory strains were used in the moonlight entrainment experiments. The Por-1SL strain (sampled in Port-en-Bessin, France, Figure S1) emerges during both full moon and new moon, therefore called semilunar (SL; see Kaiser et al., 2021 for nomenclature of timing strains). The Vigo-2NM strain (sampled in Vigo, Spain, Figure S1) emerges every new moon (NM). The strains were reared according to (Neumann, 1966), at 18 °C (±1.5 °C) and under a light-dark (LD) cycle of 16:8 h with a 4000 K neutral white LED light (61001491201 from Hera GmbH & Co. KG, Germany) in all experiments. The measured spectrum (using ILT950 spectroradiometer, International Light Technologies, Peabody, MA, USA) of the artificial daylight is given in Figure S5a. The moonlight provided varied depending on the experiment (see below).

For setting up experimental boxes, fertilized egg clutches collected over 4 days from several culture boxes were de-jellied with 5% bleach for a minute and rinsed five times with pasteurized 50:50 seawater and deionized water. Around 250 eggs were placed in transparent plastic boxes (8 × 8 × 8 cm) with 160 mL of pasteurized water (50:50 seawater and deionized water) and 2 g of sand collected in different natural habitats of *C. marinus* (France and Portugal). Larvae were fed twice a week with 2 mL of diatoms (*Phaeodactylum tricornutum*, strain UTEX 646). The water was exchanged every 2 weeks and after that the larvae were supplied with powdered nettles (*Urtica* sp., Phoenix, Pharmahandel GmbH & Co. KG, Germany). For one experiment, the simulated natural moon light with 6 h high intensity, the boxes were set up differently: 20 fertilized egg clutches (around 1000 eggs) were washed in deionized water for 1 h and placed in larger plastic boxes (20 × 20 × 5 cm). Larvae were fed twice a week with 5 mL of diatoms and the water was exchanged as mentioned above.

Emergence phenotypes were obtained by counting the number of adults every day in the morning or early afternoon, over a minimum of two moonlight cycles and in three to six replicate boxes. Both strains emerge typically in late afternoon or night, so that the emergence day was recorded as the day before collection. The total number of individuals per cycle per treatment and replicate is given in Table S2. The fraction of emerged midges per experiment is given in Table S3 and Table S4.

Experimental boxes were placed in acclimatized rooms or in an I36-LL Percival incubator (Percival Scientific, USA). Light and temperature were measured over the course of the experiments with HOBO Pendant data loggers (UA-002-08 or UA-002-64, Onset Computer Corp, USA). A Q203 Quantum PAR Radiometer (Irradian Ltd., UK) was used to characterize in detail irradiance (W/m^2^) and illuminance (lux) of the moonlights tested in this study (Table S5).

### Moonlight Entrainment Experiments

#### Experiment 1: Moonlight Intensity

**Figure 2.**
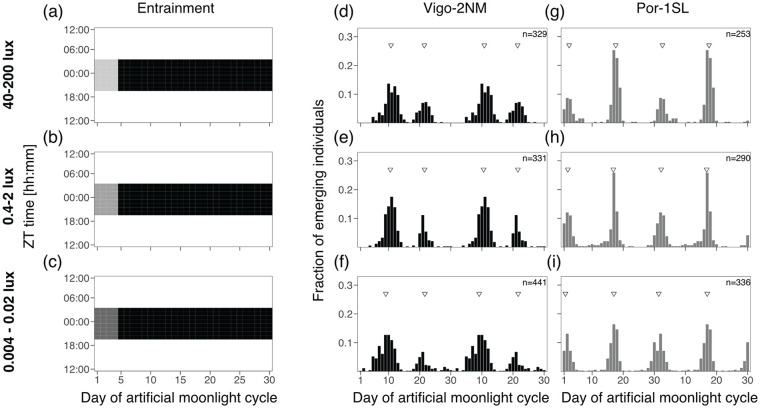
Moonlight intensity does not set the phase of the semilunar or lunar rhythm. **(a-c)** Artificial moonlight was provided throughout the night (8 h) for 4 consecutive nights in a 30-day artificial moonlight cycle. Three light intensities were tested **(a)** 40-200 lux, **(b)** 0.4-2 lux and **(c)** 0.004-0.02 lux (Table S5). Heatmaps illustrate the moonlight treatment, where the x-axis represents days of moonlight cycle and y-axis shows zeitgeber time (ZT). Day 1 of the moonlight cycle is considered the first day moonlight is provided. Daylight is depicted in white, night in black and the artificial moonlight in different shades of gray. Emergence of the strains Vigo-2NM **(d-f)** and Por-1SL **(g-i)** are shown in barplots. Fraction of emerging individuals (y-axis) is shown over the moonlight cycle (x-axis), double-plotted for visualization. Total number of individuals per strain (values on top right) correspond to several replicate boxes over consecutive cycles summed up (Table S2). Inverted triangles show the phase of the rhythm detected with CircMLE (see Methods, Table S6). Differences in phase of entrainment are marginal across treatments for both strains (compare triangles).

In order to test if moonlight intensity affects the strength or phase of entrainment, we entrained the strains with different light intensities. We started from the historical standard treatment, which is moonlight presented throughout the night for four consecutive nights every 30 days (Figure 2). Day 1 in the moonlight cycle was considered to be the first day the strains were subjected to light at night. Different moonlight intensities were simulated using white LED Array lights (LIUCWHA from Thorlabs, USA) dimmed with different neutral density filters (NDUV10B, NDUV20B and NDUV30B, Thorlabs, USA) to consistently decrease light intensity: (1) NDUV10B nominal optical density (OD) of 1, (2) NDUV30B with OD3 and (3) NDUV20B and NDUV30B in combination to obtain OD5. Light intensity across the spectrum (Figure S5b) was measured using a ILT950 spectroradiometer (International Light Technologies, Peabody, MA, USA). Total light intensity (Table S5) was measured by a Q203 Quantum PAR Radiometer (Irradian Ltd., UK). Depending on the position in the experimental setup, the light intensities were 40-200 lux for OD1, 0.4-2 lux for OD3 and 0.004-0.02 lux for OD5. As these intensity values were not obtained from measurements underwater, it might be that light intensity reaching the developing larvae is lower than these ranges. In previous reports, the standard treatment was presented at intensities similar to OD3 (Kaiser et al., 2011; Neumann, 1966) or in recent years rather OD1 (Kaiser et al., 2021). Therefore, we use the standard treatment at OD1 as a comparison for all other experiments. In nature, moonlight intensity during full moon is expected to be between 0.05 and 0.2 lux (Kyba et al., 2017), that is, in between treatments OD3 and OD5.

#### Experiment 2a and 2b: 2- and 4-h Sensitivity Windows

**Figure 3.**
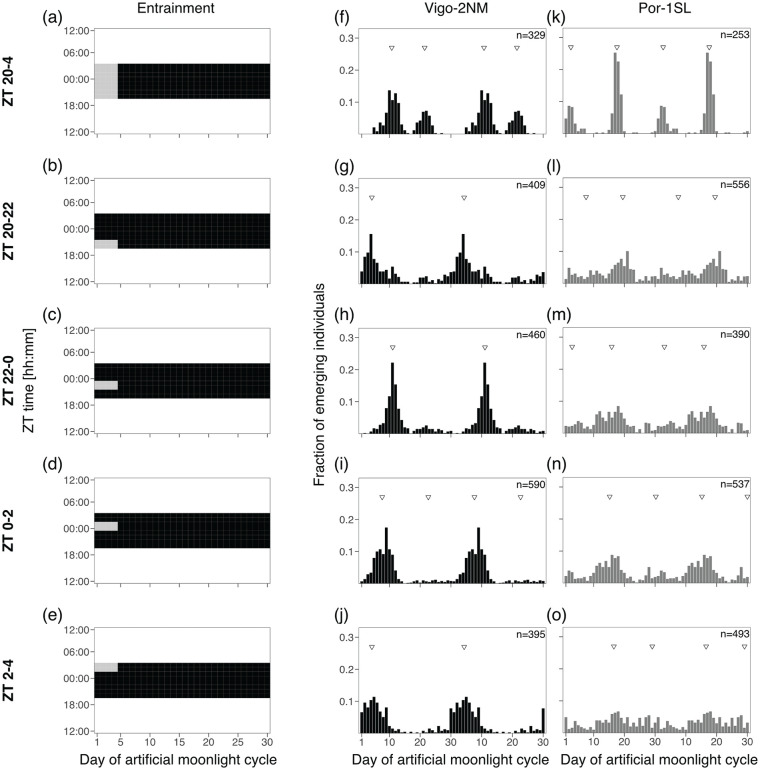
Two hours of light at various times of the night entrain the rhythm in the Vigo-2NM strain but not in the Por-1SL strain. **(a-e)** A block of 8 h of artificial moonlight (standard treatment) was compared with 2 h of artificial moonlight over 4 days at different times of the night (light gray rectangles). **(a)** Standard treatment of 8 h of light at night (data from Figure 2), **(b)** moonlight from ZT 20-22, **(c)** moonlight from ZT 22-0, **(d)** moonlight from ZT 0-2 and **(e)** moonlight from ZT 2-4. Heatmaps illustrate the different entrainments tested, where the x-axis shows days of moonlight cycle and y-axis shows ZT time (ZT 0 is the middle of the night). Emergence of the strains **(f-j)** Vigo-2NM and **(k-o)** Por-1SL. Barplots show the fraction of emerging individuals (y-axis) over the moonlight cycle (x-axis), double-plotted for visualization. Total number of individuals per strain (values on top right) correspond to several replicate boxes over consecutive cycles summed up (Table S2). Inverted triangles show the detected phase of the rhythm with CircMLE (see Methods, Table S6). In Vigo-2NM strain, strength of entrainment varies depending on the timing of the moonlight at night and is highest in the window ZT 20-0 (note that the artificial peak is absent). In this window, the phase of the rhythm matches the standard treatment shown in panel f (data from Figure 2d). The later the light is given at night, the earlier emergence occurs (Table S6). In contrast, none of the 2-h windows properly entrain the circasemilunar clock of Por-1SL strain (compare with panel k, data from Figure 2d, Table S6).

**Figure 4.**
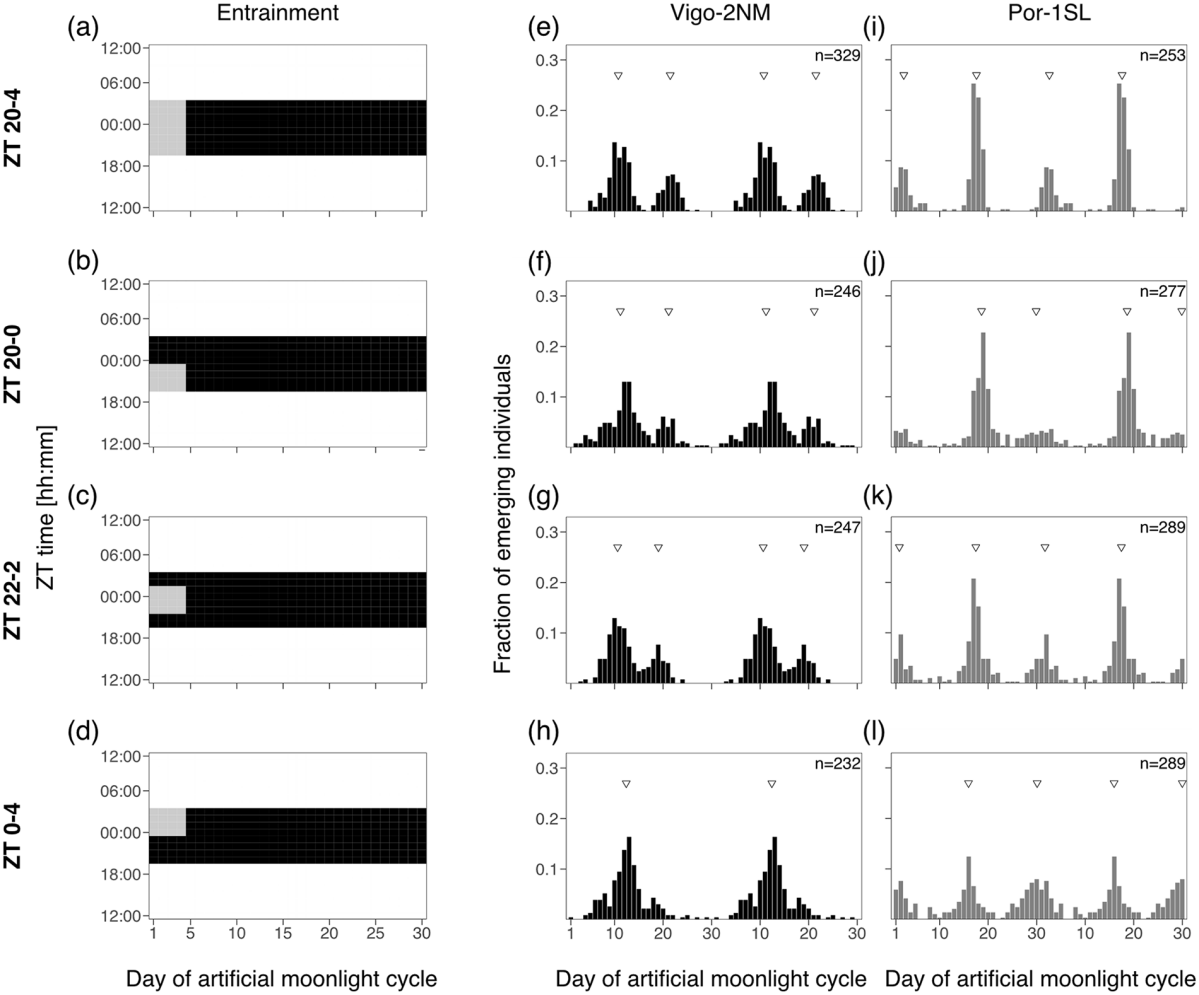
Four hours of light at various times of the night entrain the rhythm in both strains. **(a-d)** A block of 8 h of artificial moonlight (standard treatment, Figure 2a) was compared with 4 h of artificial moonlight over 4 days at different times of the night (light gray rectangles). **(a)** Standard treatment of 8 h of light at night (data from Figure 2a), **(b)** moonlight from ZT 20-0, **(c)** moonlight from ZT 22-2 and **(d)** moonlight from ZT 0-4. Heatmaps illustrate the different entrainments tested, where the x-axis shows days of moonlight cycle and y-axis shows ZT time (ZT 0 is the middle of the night). Emergence of the strains **(e-h)** Vigo-2NM and **(i-l)** Por-1SL is shown. Barplots show the fraction of emerging individuals (y-axis) over the moonlight cycle (x-axis), double-plotted for visualization. Total number of individuals per strain (values on top right) correspond to several replicate boxes over consecutive cycles summed up (Table S2). Inverted triangles show the detected phase of the rhythm with CircMLE (see Methods, Table S6). All 4-h windows entrain Vigo-2NM and Por-1SL strains but strength of entrainment is lower in Vigo-2NM (compare with standard treatment and 2-h window ZT 22-0 in Figure 3h). In Por-1SL strain, strength of entrainment is high for all windows (Table S6). In Vigo-2NM strain, the windows ZT 20-0 and ZT 22-2 match the phase of the standard treatment whereas in Por-1SL strain the match is only observed for window ZT 22-2.

In order to test if there are specific windows of light at night for which the two *C. marinus* strains are exclusively sensitive to moonlight, windows of 2 or 4 h over four consecutive nights were provided every 30 days. Day 1 of the moonlight cycle was considered as above. Zeitgeber time (ZT) 0 is defined as the middle of the night. For the 2-h window experiment (Figure 3), four discrete windows were tested over the 8 h of darkness: ZT 20-22, ZT 22-24, ZT 24-2 and ZT 2-4. Moonlight was mimicked with a 4000 K neutral white LED light (VT-2216, 7492, Pollin Electronic GmbH, Germany), with light intensity around 325 lux (Table S5). For the 4-h window experiment (Figure 4), three overlapping windows were tested: ZT 20-24, ZT 22-2 and ZT 0-4. Moonlight was provided with a White LED Array lights (LIUCWHA from Thorlabs, USA) with a NDUV10B filter and light intensity 40-200 lux (Table S5).

#### Experiment 3: Shifting Block of Moonlight

**Figure 5.**
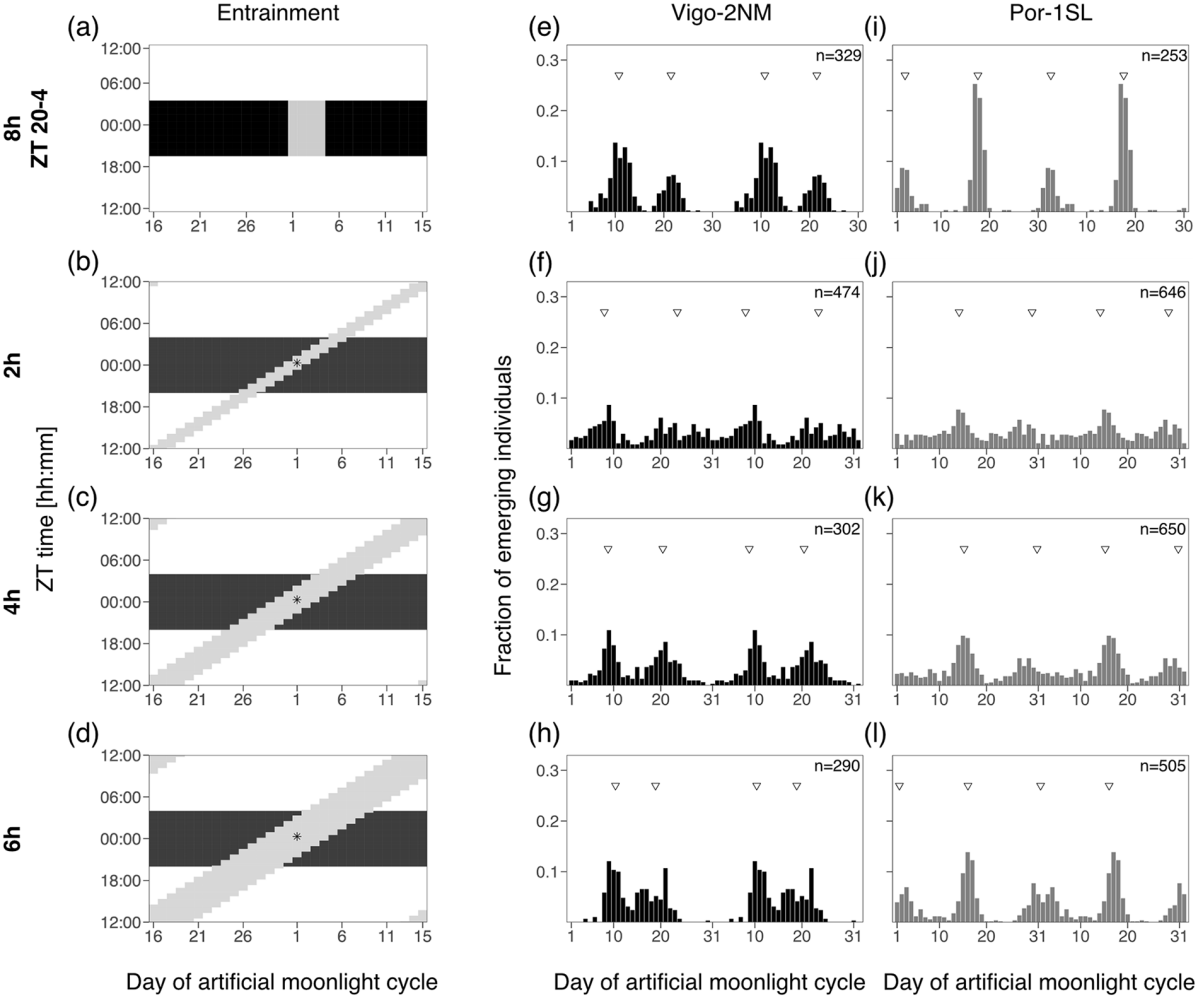
Duration of a shifting block of moonlight impacts the strength of entrainment of the circa(semi)lunar clock. **(a-d)** A block of 8 h of artificial moonlight (standard treatment, Figure 2a) was compared with moonlight presented in a lunar day cycle (24.8 h), that is, with a daily shift of 48 min of a high-intensity moonlight (40-200 lux, light gray shading). The daily shift of 48 min results in a moonlight cycle of 31 days (x-axis). Y-axis shows ZT time. Day 1 in all entrainments was considered the day where the middle of the moonlight occurs at ZT 0, the middle of the night (asterisk). Moonlight duration was **(b)** 2 h, **(c)** 4 h or **(d)** 6 h. Emergence of the strains **(e-h)** Vigo-2NM and **(i-l)** Por-1SL. Barplots show the fraction of emerging individuals (y-axis) over the moonlight cycle (x-axis), double-plotted for visualization. Total number of individuals per strain (values on top right) correspond to several replicate boxes over consecutive cycles summed up (Table S2). Inverted triangles show the detected phase of the rhythm with CircMLE (see Methods, Table S6). The 2-h shifting block is insufficient to properly entrain the circa(semi)lunar clock of Por-1SL or Vigo-2NM strains. The phase of the rhythm under 6- and 4-h shifting blocks matches the standard treatment in both strains. This suggests that the strains are most sensitive to moonlight in the middle of the night.

In *C. marinus’* natural habitat, moonlight intensity is a complex environmental cue that depends on the lunar phase and on the tidal cycle. Every day, moonrise and moonset shift by about 48 min. Likewise, tidal cycles have a period of 12.4 h and high-tide and low-tide shift every day by about 48 min and can hinder moonlight visibility (see above and discussion). Here, we tested if the natural timing of moonlight alone (without intensity changes) affects the entrainment of the circalunar clock. Changing only one aspect at a time, we also used the high moonlight intensity of the standard treatment. Therefore, we simulated moonlight as a block of light (2, 4 or 6 h duration) shifting every day by 48 min. This resulted in a cycle of 31 days with 30 moonlights (Figure 5). Moonlight was simulated using white LED Array lights (LIUCWHA from Thorlabs, USA) together with a neutral density filter NDUV10B (intensity 40-200 lux, Table S4) connected to an Arduino Mega 2560 microcontroller (Arduino, Italy, http://www.arduino.cc/). Custom-made scripts (available at https://doi.org/10.17617/3.6OXUES) were written to turn the lights on and off with the duration of a lunar-day, that is, with a daily shift of 48 min. The intrinsic error rate of each Arduino was assessed by measuring the duration of light transitions over consecutive days with HOBO Pendant (measurements each 10 sec, data not shown). This error was then taken into consideration in each loop run by the Arduino in order to obtain the expected timings. To match the emergence patterns across treatments, we considered Day 1 as the day when the middle of the moonlight treatments occurs at ZT0 (asterisks in Figure 5).

#### Experiment 4: Simulated Natural Moonlight

**Figure 6.**
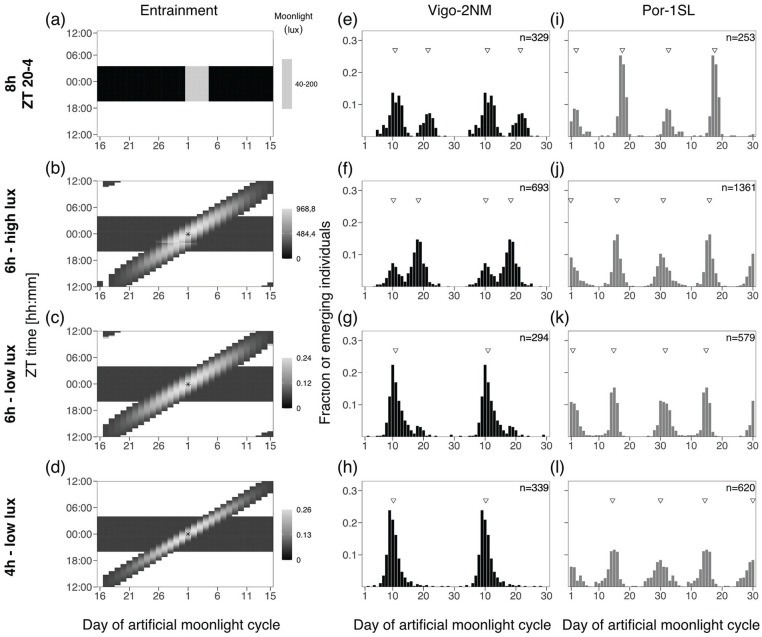
Simulated natural moonlight at natural intensities entrains both strains well. **(a-d)** A block of 8 h of artificial moonlight (standard treatment, Figure 2a) was compared with moonlight presented in a lunar day cycle (24.8 h), that is, with a daily shift of 48 min together with light intensity modulation. Moonlight intensity was changing gradually over the night and over the lunar cycle. Heatmaps are colored-scaled for light intensity throughout the 30-day moonlight cycle (x-axis) and ZT time (y-axis). Day 1 in the simulated natural moonlight treatments was considered the day where the middle of the moonlight occurs at ZT 0, the middle of the night (asterisk). **(b)** High-intensity moonlight (similar to the standard treatment, ~900 lux) was tested with a duration of 6 h. Natural levels of moonlight during full moon (< 0.3 lux) were tested with a duration of **(c)** 6 h and **(d)** 4 h. Emergence of the strains **(e-h)** Vigo-2NM and **(i-l)** Por-1SL. Barplots show the fraction of emerging individuals (y-axis) over the moonlight cycle (x-axis), double-plotted for visualization. Total number of individuals per strain (values on top right) correspond to several replicate boxes over consecutive cycles summed up (Table S2). Inverted triangles show the detected phase of the rhythm with CircMLE (see Methods, Table S6). In Vigo-2NM strain, both 4- and 6-h low-intensity light treatments entrain the circalunar clock without artificial peak. The 6 h treatment of high intensity leads to a strong artificial peak that mirrors the observed emergence pattern in the 6 h-shifting block. In Por-1SL strain all simulated moonlight treatments lead to a strong entrainment of the circasemilunar clock.

In this experiment, we simulated both the natural timing of moonlight (i.e., the daily shift), as well as changing moonlight intensity over the night and over the lunar cycle. This was achieved with a Profilux Light computer (GHL Advanced Technology GmbH & Co. KG, Germany) with a customized firmware. The computer was connected to a dimmable (by pulse width modulation) LED Mitras Lightbar 2 Actinic 120 (PL-1294, GHL Advanced Technology GmbH & Co. KG, Germany), using cold white (8000 K) color channel and a maximum light intensity set to 100% (measured spectrum in Figure S6a). Briefly, the light program consists of a sine wave of light intensity applied to a 30-day cycle to simulate lunar phases. Then, a second sine wave of light intensity is applied to the 24 h light cycle, simulating the rise and fall of the moon or the effect of water levels respectively. The day with maximum moonlight intensity across the lunar cycle is defined as full moon and on that day, the daily maximum moonlight intensity is set to the middle of the night (asterisks in Figure 6). This was considered as Day 1 of the moonlight cycle. Maximum light intensity shifts every day for 48 min simulating the 24.8-h lunar day. The width of the daily sine wave (4 or 6 h) corresponds to time length of light provided to further simulate the effect of the water levels on light visibility. We applied three different treatments: First, only introducing a single change, we inserted dimming over the daily cycle but kept a high maximal intensity of moonlight similar to the standard treatment. The treatment therefore was a 6-h moonlight of high intensity (average maximum intensity around 900 lux, Table S5). Second, we then reduced the maximum intensity to natural moonlight intensities, resulting in a 6-h moonlight of low intensity (average maximum intensity around 0.24 lux, Table S5). Finally, we mimicked the light restricting effect of the tides by shortening the moonlight duration to 4 h, still at low intensity (average maximum intensity around 0.26 lux, Table S5). Because the computer program calculates light intensities per day and fits the 48-min shift to a 30-day cycle, there is a jump in light intensity at midnight for the 6-h high-intensity moonlight treatment (see Figure 6d and Figure S6b). After we had spotted this shortcoming in the 6-h high-intensity moonlight treatment, for the other treatments, the recalculation was set to occur during the day and thus moonlight during the night was not affected (Figure S6c).

For the low light intensity treatments, light intensity was reduced by covering the lightbar with aluminum foil, punctured evenly over its length. The lightbars were placed at the back of I36-LL Percival incubators (Percival Scientific, USA) and illuminance (Table S5) was measured using a Q203 Quantum PAR Radiometer (Irradian Ltd., UK) to ensure all replicate boxes were exposed to the same light intensity.

### Matching Phase-Setting Moonlight Windows in the Laboratory and the Field

**Figure 7.**
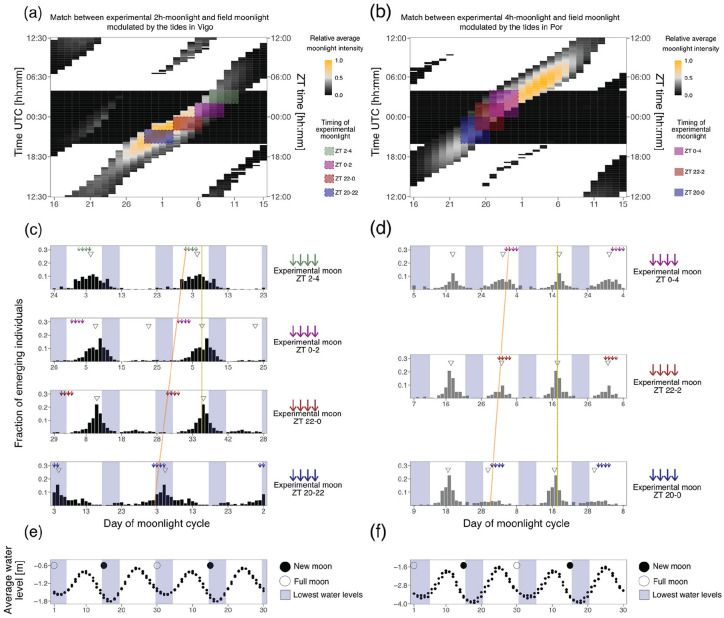
Phase-shifts due to timing of light at night might be adaptive and match the natural timing of emergence of the Vigo-2NM strain. **(a-b)** Heatmaps of simulated moonlight (water levels multiplied with relative moonlight intensity). Only 4 h of light around low tides are shown. Daylight is shown in white and night in black. Days in the moonlight cycle are shown on the x-axis, the time in UTC is shown on the left y-axis and ZT time is shown on the right y-axis. The field light-dark cycle was fitted to a 16:8 LD cycle and matched with the 16:8 LD cycle from the experiment based on the middle of the night. Colored boxes represent the tested sensitivity windows for **(a)** the Vigo-2NM strain under 2 h moonlight windows and **(b)** the Por-1SL strain under 4 h moonlight windows. The windows were matched to the field data so they had maximal overlap with the simulated moonlight for four consecutive nights. For the Vigo-2NM strain, this results in ZT 20-22 matching Day 29-2 in field (blue rectangle), ZT 22-0 match Day 3-6 (red rectangle), ZT 0-2 match Day 6-9 (pink rectangle) and ZT 2-4 match Day 8-11 (green rectangle). For the Por-1SL strain, ZT 20-0 matches Day 23-26 in field (blue rectangle), ZT 22-2 matches Day 25-28 (red rectangle) and ZT 0-4 matches Day 27-30 (pink rectangle). **(c)** Emergence of Vigo-2NM strain under 2 h-windows at different times of the night. Emergence data from each tested window in Figure 3 was replotted and shifted according to when this window is expected to occur in the field (see panel a). Arrows represent days in which the moon was provided and are colored by the 2 h-window tested. The orange line indicates the shift in the predicted days of moonlight. Inverted triangles show the detected phase of the rhythm with CircMLE (see Methods, Table S6). The phase is not shifting, as is indicated by the yellow line. Blue shadings represent the spring tides predicted in (**e**). Under most windows, emergence occurs just before the new moon spring tides, as is generally observed for new moon populations. Thus, the experimentally observed shifts in phase depending on the moonlight windows would under natural conditions allow emergence to happen at the same time of the lunar cycle. **(d)** Emergence of Por-1SL strain under 4 h-windows at different times of the night. Again, the observed shift in phase of emergence (relative to the orange line) ensures that emergence happens at the same time of the lunar cycle (yellow line). **(e-f)** Averaged low tide water levels (y-axis) and respective times of new moon (black circle) and full moon (white circle) are shown in a 30-day cycle (x-axis; double-plotted for visualization). Blue shadings represent the 4 days with the lowest water levels, that is, the spring tides.

We further explored how the phase-shifts detected in Experiment 2a and 2b relate to the optimal adult emergence timings in the field. First, a “Vigo moon*tides 4 h data set” and a “Por moon*tides 4 h data set” were generated by filtering the respective “simulated underwater” data set (see above; before averaging months) to allow the light to be visible for only 2 h before and after low tide. The data set was then subsetted into lunar cycles (Day 1 = full moon) and averaged into one representative 30-day cycle (Figure 7a and 7b). The experimentally tested entrainment windows in experiments 2a and 2b were fitted into the moonlight windows of the “Vigo moon*tides 4 h data set” and “Por moon*tides 4 h data set” (colored boxes in Figure 7a and 7b). For this fitting, the daylight timing in the field (Figure 7a and 7b, left y-axis) and the LD cycle from the experiment (Figure 7a and 7b, right y-axis) needed to be matched. To this end, from the R package “suncalc” we retrieved the sunrise and sunset values for the longest day during the eight consecutive months which were assessed. In Vigo, this resulted in a LD of 15:9 which was fitted in a LD 16:8, with the light phase between 0430 and 2030. In Port-en-Bessin, the LD was 16:8 with the light phase being between 0400 and 2000. Based on this, the middle of the dark-phase was matched at ZT0 (Vigo at 0030 UTC and Port-en-Bessin 0000 UTC). Then, each experimental 2-h or 4-h light window was placed on the field days for which there were at least 2 h of moon visible in four consecutive days. This matching translates into a prediction of which days the observed emergence times in the laboratory would fall to in the field (Figure 7e and 7f). Finally, we compared these predictions to the optimal timing, that is, the spring tide days. To this end, water levels during low tides (see section “Inferring light visibility in the intertidal of different geographical locations”) were averaged to indicate low tide levels (dots in Figure 7c and 7d) and the corresponding spring tide days (the lowest low tide of the month ±2 days, blue shading in Figure 7c to 7f).

### Statistical Analysis

#### Phase and Peak Concentration From CircMLE

Phase and peak concentration were determined using the statistics software R (version 4.2.3) and the package CircMLE (Fitak and Johnsen, 2017). CircMLE uses a likelihood-based approach to analyze unimodal or bimodal circular data according to 10 defined models. Briefly, the uniform model (M1) has no significant directionality (arrhythmicity), unimodal models (M2A, M2B, M2 C) have a single direction (single emergence peak) and bimodal models (M3A, M3B, M4A, M4B, M5A, M5B) have two significant directions on the circle (two emergence peaks). Each model is described by parameters such as mean direction (φ1 and φ2) and concentration (k1 and k2), which in chronobiology approximates the phase and peak concentration, respectively. We considered φ1 as the phase of the peak detected closer to Day 1 (Peak 1, Table S6) and φ2 as the phase of the second detected peak (Peak 2, Table S6). The likelihood that each data set fits into each of the 10 models was evaluated, and the most likely model was selected according to the Akaike Information Criterion (AIC). AIC is the sum of the model fit (deviance) and twice the number of parameters. It penalizes models with more parameters, thus the model with the lowest AIC was considered the most suitable (Table S6). We run CircMLE with no a priori assumption on the expected emergence distributions and considered all provided models to fully examine the effects of the light treatments.

#### Rhythmicity Index (RI)

In order to assess the strength of entrainment, we calculated the rhythmicity index (RI) for each experimental treatment. RI was calculated according to (Levine et al., 2002) with a few modifications. In short, number of emerged individuals per day was first normalized per cycle (30 days) and autocorrelation was calculated using *acf* function of *tseries* R package v0.10-55 (Trapletti and Horni, 2023). RI represents the autocorrelation function of a lag of 16 for semi-lunar populations (period of 15 days) and of a lag of 31 for lunar populations (period of 30 days). Significance of the rhythm is descriptively defined as RI > 0.3 = highly significant, 0.1 < RI < 0.3 = significant, RI < 0.1 = not significant.

## Results

### Moonlight Intensity Is Modulated by the Tides

We measured light intensities in the intertidal zone of Dinard with a hyperspectral radiometer placed 1 m below the low water of the theoretical maximal tidal range. Plotting out the raw data (Figure S2a) shows a clear modulation of daylight across the lunar cycle, with light intensities strongly increasing during the spring tide days around full moon and new moon. There is also some day to day variation which likely comes from the weather conditions (Figure S2a). The measured spectrum of daylight is given in Figure S2b. Moonlight intensity is so low that it cannot be seen in the raw data plot (Figure S2a) and the spectrum obtained for moonlight is very noisy (Figure S2c). In order to decrease the noise, we averaged the data in 30 min bins and summed up the values in wavelength categories (300-399 nm, 400-499 nm, etc.; see Methods). We then plotted light intensity in heatmaps which separate the lunar cycle time axis (x-axis) and the daily time axis (y-axis). We found that the intensity of both moonlight and daylight is also modulated by the tides along the daily time axis (Figure 1a, Figure S3, Figure S4). Light intensities were the highest during low tide for both moonlight (Figure 1a) and daylight (Figure S4). The 50-min shift in the highest daily moonlight intensity following the 24.8-h lunar-day can be observed, as well as the increasing and decreasing light intensity toward and after full moon days (Figure 1a).

The highest intensities of both moonlight and daylight are found at wavelengths between 400 and 700 nm (Figure S2b and S3a and c). Normalizing daylight intensity for each wavelength category independently (Figure S3d) reveals that daylight is also detectable at wavelengths below 400 nm and above 700 nm and that tidal modulation of light is relatively uniform over the measured spectrum (Figure S4). However, when normalizing moonlight intensity for each wavelength category independently (Figure S3b), it is not possible to distinguish the signal of moonlight from background noise at wavelengths other than 400-700 nm (Figure S3a-b). This shows that moonlight can only be effectively detected by the radiometer in the range of 400-700 nm.

Daylight was detected during every day-time low tide (Figure S3c). In contrast, moonlight is primarily detected during the night-time low tides around full moon (Figure S3a). During new moon there are also traces of light at night, but much less intense than during full moon (Figure S3a), suggesting that the low ambient light during new moon is still modulated by the tides.

In order to explore by how much the water levels restrict the availability of moonlight, we compared the light measurements we obtained from the intertidal zone (Figure 1a) with calculated moonlight intensities in-land (Figure 1b). While relative moonlight illumination over Dinard during full moon can last up to 14 h (Figure 1b), in the measurements from the intertidal zone it is only detectable above background noise for approximately 4-5 h (Figure 1a).

In each geographical location, the timing of the tides varies. Consequently, the timing of light at night and the lunar phase during which highest light intensities reaching *C. marinus* larvae may differ. We tested if combining the publicly available data sets of in-land moonlight and water levels in Dinard would recapitulate the moonlight patterns observed in underwater measurements. There is a good correspondence between the calculated patterns (Figure 1c) and the field measurements (Figure 1a). Therefore, we used this simulation to predict the patterns of moonlight visibility at the sites where *C. marinus* strains used in entrainment experiments described below were sampled (Figure 1d and 1e). In Dinard, the days with low tides in the middle of the night correspond to full moon days (Figure 1c). However, this is not the case for other locations along the coast. Therefore, the patterns differ from those found in Dinard in three aspects (compare Figure 1c to Figure 1d and 1e). First, moonlight available in the middle of the night occurs several days before full moon (Por; Figure 1e) or after full moon (Vigo; Figure 1d). Second, the highest moonlight intensity around full moon is late in the night (Por; Figure 1e) or early in the night (Vigo; Figure 1d). Third, some low-intensity moonlight may also be visible in a second window just before new moon (Por; Figure 1e) or just after new moon (Vigo; Figure 1d).

In entrainment experiments, which are described below, we tested how intensity, duration and time of light at night affect the strength and phase of entrainment and how adult emergence matches the timing of lowest tides based on the prediction when *C. marinus* strains would perceive light at night in their respective geographical location.

### Experiment 1: Moonlight Intensity Does Not Set the Phase of the Semilunar or Lunar Rhythm

Four consecutive nights with dim illumination were previously established as the standard artificial moonlight treatment for entrainment of *C. marinus’* circalunar clock (Neumann, 1966).

Here, we first asked if exposing the strains Vigo-2NM and Por-1SL to different moonlight intensities under the standard treatment would affect the strength or phase of entrainment. A light set up with optical density filters was used to provide three independent treatments, namely, 40-200 lux (Figure 2a), 0.4-2 lux (Figure 2b) and 0.004-0.02 lux (Figure 2c).

We found that phase of entrainment is unaffected by light intensity in both Vigo-2NM (Figure 2d to 2f) and Por-1SL (Figure 2g-i) strains. Across treatments, both Peak 1 and Peak 2 differ in phase by less than a day (Table S6).

Notably, lower light intensities do not reduce the strength of entrainment in Vigo-2NM (Figure 2f) and Por-1SL (Figure 2i) strains, as observed in the Rhythmicity Index RI (Table S6). This confirms that *C. marinus* is exquisitely sensitive to moonlight down to at least 0.004 lux.

The two emergence peaks of the Por-1SL strain are of unequal size under high-intensity treatments (Figure 2g and 2h). However, we think the inequality is not necessarily due to the light treatment, but rather due to a developmental effect: In these experiments most individuals were developmentally ready to emerge at approximately the same time, which lead to some emergence peaks being much stronger than others, which translates into an inequality of the peaks in the summed histograms.

In all treatments, the Vigo-2NM strain shows a smaller second emergence peak, about 10 days after the first peak and not fitting a semi-lunar pattern. The second peak is slightly stronger at higher light intensities (Figure 2d vs 2f). This peak is considered an artifact of the highly artificial moonlight regime, as it is absent in the field and also under more realistic moonlight regimes in the laboratory (Kaiser et al., 2021). The experiments below also explore which moonlight regimes lead to an emergence pattern without the artificial peak.

### Experiment 2a: 2 h of Light at Various Times of the Night Entrain the Rhythm in the Vigo-2NM Strain but Not in the Por-1SL Strain

Next, we explored (1) if a moonlight sensitivity window as described for the San strain (Neumann, 1969) also exists in the Por-1SL and Vigo-2NM strains, if (2) its duration and/or timing in relation to the light-dark cycle is strain-specific, and if (3) it sets the phase of the circalunar clock. Taking the standard treatment at high intensities (OD1) as a starting point (Figure 3a, 3f, and 3k; replotted from Figure 2a, 2d, and 2g), we only changed the duration of moonlight and exposed these strains to a bright artificial moonlight in 2-h windows. We applied four independent treatments consisting of windows corresponding to “after dusk” (ZT 20-22, Figure 3b), “middle of the night” (ZT 22-0, Figure 3c and ZT 0-2, Figure 3d) and “before dawn” (ZT 2-4, Figure 3e).

We found that 2 h of light at night entrain the circalunar clock of the circalunar strain Vigo-2NM. Strength, rhythmicity and phase of entrainment differ depending on the timing of light at night (Figure 3g to 3j). Shorter moonlight durations clearly reduce the strength of the artificial peak. Except for the window ZT 0-2, where the model that best describes the emergence distribution is bimodal (M4B, Table S6), unimodal distributions were detected (as expected for a circalunar strain). Depending on the timing of moonlight, the strength of entrainment varies greatly (see RI, Table S6). The window ZT 22-0 (Figure 3h) not only leads to the strongest peak concentration and highest RI (Table S6), but also the phase of the emergence patterns matches the standard treatment (compare Figure 3h with 3f). This suggests that the Vigo-2NM strain is most sensitive to moonlight during the 2 h prior to the middle of the night. Providing light at night 2 h after the middle of the night (ZT 0-2) leads to an advance in the phase of emergence of around 3 days compared to the standard treatment. Interestingly, moonlight windows after dusk (ZT 20-22; Figure 3g) and before dawn (ZT 2-4; Figure 3j) windows lead to a phase advance of around 6 days, but strength of entrainment is low (see RI, Table S6).

In contrast, for the Por-1SL strain we found that 2 h of light at night do not properly entrain the circasemilunar clock (Figure 3l to 3o). In all treatments, there are individuals emerging throughout the lunar cycle, which is not observed in the standard treatment (compare Figure 3k with 3l to 3o). CircMLE detects bimodal distributions for all treatments but with low concentration peaks (on average less than 2, Table S6) and only the window from ZT 0-2 had a significant RI (Table S6).

### Experiment 2b: 4 h of Light at Various Times of the Night Entrain the Rhythm in Both Strains

As the Por-1SL strain was not entrained by 2 h of light at night, we tested if increasing the duration of light to 4 h would entrain its circasemilunar clock. Alongside, we also tested this treatment in the Vigo-2NM strain. We used three different windows of light at night, ZT 20-0 (“after dusk,” Figure 4b), ZT 22-2 (“middle of the night,” Figure 4c) and ZT 0-4 (“before dawn,” Figure 4d), and compared to the standard treatment (Figure 4a, 4e, and 4i, replotted from Figure 2a, 2d, 2g).

In the Vigo-2NM strain, 4 h of light at night lead to less concentrated peaks in most treatments compared to 2 h of light at night (Figure 4f to 4h compared with Figure 3h). For the 4 h moonlight windows most models suggest a bimodal distribution, showing that this entrainment is less optimal for Vigo-2NM strain as it increases the artificial peak around Day 20 (see k2, Table S6). The different treatments entrain the rhythm in slightly differing phases (-1 to -2 days, see Peak 1 in Table S6). The window in the middle of the night (ZT 22-2, Figure 4g) sets the phase similarly to the standard treatment. The window after dusk (ZT 20-0, Figure 4f) induces a delay of less than a day while the before dawn window leads to a phase delay of almost 2 days (ZT 0-4, Figure 4h) with a strong strength of entrainment (RI = 0.38, Table S6).

We found that in the Por-1SL strain, 4 h of light at night are sufficient to entrain the circasemilunar clock (Figure 4j to 4l). In all treatments, emergence displays a bimodal distribution (M5B, Table S6). There are some differences in the concentration of peaks across treatments, with the moonlight window in the middle of the night (ZT 22-2, Figure 4k) leading to the strongest rhythmicity (RI = 0.35, Table S6). The emergence distribution under this treatment also most closely matches the standard treatment (compare Figure 4k with 4i). The phase of emergence changes slightly with the timing of the light window (Figure 4j to 4l; Table S6). Taken together, these results suggest that the Por-1SL strain requires longer moonlight durations than the Vigo-2NM strain and is most sensitive to moonlight in the middle of the night.

### Experiment 3: Duration of a Shifting Block of Moonlight Impacts the Strength of Entrainment of the Circa(Semi)Lunar Clock

With the simplified artificial moonlight regimes tested up to here, we were able to show that (1) moonlight intensity does not affect the phase or strength of entrainment, (2) duration of light at night required to entrain the rhythm is strain-specific, (3) light presented in the middle of the night sets the phase similar to the standard moonlight treatment, and (4) the timing of light at night usually shifts the phase of the lunar rhythm. However, moonlight is a complex environmental cue, with intensities changing across the lunar cycle and due to the effect of the tides (Figure 1). Therefore, we next tested a shifting-block moonlight treatment which simulates the daily shift of the moonlight onset and offset of 48 min under a 31-day cycle. Changing only one aspect at a time, we kept a high intensity of moonlight (40-200 lux, as the standard treatment). The effect of the tides hindering moonlight visibility was simulated by changing the duration of the block of light: 2 h (Figure 5b), 4 h (Figure 4c) or 6 h (Figure 5d).

The 2-h shifting block of moonlight neither entrained the circalunar clock in Vigo-2NM strain (Figure 5f) nor the circasemilunar clock in Por-1SL strain (Figure 5j), even though in Vigo-2NM a non-shifting 2-h window of moonlight entrained the clock effectively (Figure 3g to 3j). The 4- and 6-h shifting-block treatments lead to reasonable entrainment in the Vigo-2NM strain (Figure 5g and 5h, respectively) and the Por-1SL strain (Figure 5k and 5l, respectively). However, in the Vigo-2NM strain the artificial peak is particularly pronounced and bimodal distributions were detected (Table S6), suggesting that as in Experiment 2b the longer moonlight (4 h and 6 h) increases the artificial peak.

In the shifting-block treatments, we defined Day 1 as the day that the middle of the moon hits the middle of the night (Figure 5b to 5d, asterisk). Under this definition of Day 1, the phase of emergence in the shifting-block moonlight and in the standard treatment (Figure 5a, 5e, and 5i) are very similar (with deviations of maximum 2 days, Table S6). This underlines the findings from Experiment 2a and 2b, which suggested that moonlight is best perceived in the middle of the night.

### Experiment 4: Simulated Natural Moonlight at Natural Intensities Entrains Both Strains Well

Finally, we took into account the modulation of moonlight intensity according to lunar phase, as well as the daily modulation of moonlight intensity. This means that moonlight shifted by 48 min every day, the moonlight intensities gradually changed within a day (moonrise and moonset simulation), and the maximum moonlight intensity changed throughout the lunar cycle (lunar phase simulation). We tested three different moonlight treatments. First, we only introduced the daily and lunar cycle dimming, but kept high maximal moonlight intensities comparable to the standard treatment. This was the “6-h high-intensity” treatment (Figure 6b), which had a daily window of 6 h of moonlight and the maximum intensity was almost 1000 lux, that is, about 5000-fold brighter than natural moonlight. In the second experiment, the “6-h low-intensity” treatment (Figure 6c), we additionally reduced the maximum light intensity to around 0.24 lux, that is, natural moonlight levels. Finally, in the “4-h low-intensity” treatment (Figure 6d), the total duration of light per day was reduced to 4 h, mimicking a narrower gating of light by the tides. The maximum light intensity was 0.26 lux.

We found that all simulated natural moonlight treatments entrain both the Vigo-2NM (Figure 6f to 6h) and the Por-1SL (Figure 6j to 6l) strain, and RI is highly significant for all treatments (Table S6). In the 6-h high-intensity treatment, the Vigo-2NM strain still has a very strong artificial peak (Figure 6f), resembling the pattern observed with a 6-h shifting block of high-intensity moonlight (Figure 5h). This suggests that despite the intensity modulation, the 6-h high-intensity moonlight is perceived as if it was a 6-h block of light moving through the night. The picture changes completely in low-intensity treatments, where the artificial peak is absent (Figure 6g and 6h). Interestingly, the 4-h low-intensity treatment, which most closely mimics the natural light regime, shows by far the best entrainment over all experiments (unimodal distribution M2A). This indicates that low light intensity and light intensity modulation matter for the Vigo-2SL strain, that is, the Vigo-2SL strain requires and integrates all aspects of this complex moonlight regime.

In contrast, the Por-1SL strain is strongly entrained by all natural moonlight treatments (Figure 6j to 6l). Light intensity does not seem to play a role in strength of entrainment. Notably, the 4-h low-intensity moonlight (Figure 6j) shows stronger entrainment than the 4-h shifting-block moonlight (Figure 5k). This hints that the modulation of light intensities throughout the lunar months and by the tides may also matter to the Por-1SL strain.

As in the shifting-block experiment, we defined Day 1 of the 30-day cycle as the day the middle of the moon occurs in the middle of the night, which also matches the day with strongest light intensities (full moon day). Under this definition, for both strains the phase of entrainment matches the standard control moonlight (Figure 6a, 6e, and 6i), underlining again that both strains seem to be most sensitive to moonlight in the middle of the night.

### Phase-Shifts Due to Timing of Light at Night Might Be Adaptive and Match the Natural Timing of Emergence of the Vigo-2NM Strain

The experiments above (Figures 2 to 6) show that there is a complex strain-specific integration of several moonlight components that precisely set the phase of entrainment. Interestingly, in the 2-h window in the Vigo-2NM strain and 4-h window in the Por-1SL strain, we observed phase-shifts depending on the time at night when light was given (Figures 3 and 4). This is interesting because in each geographic location there is a close to constant relationship between the lunar cycle and the timing of the tides. It implies that a low tide at a specific time of the night (corresponding to the 2 h/4 h windows when light was applied in experiments 2a and 2b) will always happen at a specific time of the lunar cycle (colored boxes in Figure 7a and 7b). Theoretically, moonlight will successively span through all of these sensitivity windows, but in nature weather condition might make moonlight only available in one window or the other. The question arises, if this will affect the phase of emergence. In this context, responding to the nocturnal light stimulus with different phases of emergence depending on when in the night it occurs, may ensure that the emergence always happens at the same and most suitable time of the lunar cycle, namely, around full and new moon when there are spring tides. To explore if this is true, that is, to what extent the observed phase-shifts in experiments 2a and 2b are adaptive, we matched the sensitivity windows to the days in which they are predicted to occur in the field (Figure 7a and 7b) and compared the resulting predicted emergence patterns (Figure 7c and 7d) to the days of spring tides (Figure 7e and 7f).

First, in both strains the phase-shift of the emergence rhythm compensates for the fact that light at different times of the night occurs on different days in the lunar cycle (Figure 7c and 7d). In other words, the time of emergence relative to the lunar cycle stays constant (Figure 7c and 7d, yellow lines), irrespective of whether light is perceived early in the night and thus early in the lunar cycle, or late in the night and thus later in the lunar cycle (see colored boxes in Figure 7a and 7b, which translate into the orange lines in Figure 7c and 7d). This can be considered adaptive, as it makes the phase of emergence robust to weather conditions, which may limit visibility of moonlight to certain times of the lunar cycle in an unpredictable manner.

Second, we found that in the Vigo-2NM strain emergence under three of the four 2-h windows is predicted to occur a few days before new moon spring tides (compare Figure 7c to 7d), while under one treatment there is an unexpected switch to emergence during full moon spring tides (ZT 20-22; see Figure 7c, bottom). The predicted emergence just before new moon corresponds to field observations for new moon populations. Much in contrast, in the Por-1SL strain emergence does not match the times of spring tides (compare Figure 7d to 7f), contradicting the observed emergence patterns in the field. This may indicate that moonlight entrainment alone is not sufficient for achieving an adaptive phase of emergence in the Por-1SL strain.

## Discussion

Moonlight has been shown to be an effective zeitgeber for lunar rhythms for many organisms living in the intertidal region (reviewed in Kaiser and Neumann, 2021). While moonlight has been measured in the Mediterranean Sea without tides (Poehn et al., 2022), the effect of the tides on the availability of moonlight in the intertidal zone has rarely been explored. We measured daylight and moonlight in the intertidal zone and found that moonlight is detectable at wavelengths between 400 and 700 nm (Figure S3a and b). While daylight is detectable at all measured wavelengths (317-953 nm; Figure S4), it is also most intense between 400 and 700 nm (Figure S3c). This is congruent with the fact that moonlight is sunlight reflected in the moon, both having highest intensity at blue wavelengths (400-500 nm) (Cohen et al., 2020). In coastal waters, the highest light intensity of daylight shifts into the green range (500-600 nm) (Mascarenhas and Keck, 2018). The same is true for moonlight, as shown in our measurements (Figure S3a). It remains unclear, if the absence of detectable moonlight below 400 nm and above 700 nm, which was also found in previous measurements of moonlight under water (Poehn et al., 2022), is due to effective filtering of moonlight by the water column, or due to sensitivity limitations of the photometer.

Our measurements of light in the intertidal zone also show a clear modulation of daylight intensity across the lunar cycle, with highest intensities detected during the spring tide days (Figure S2a). Such a modulation is not observed in light measurements from the Mediterranean, where there are no tides (Veedin Rajan et al., 2021). In addition, during each day the highest intensities of both daylight (Figure S4) and moonlight (Figure 1a) are not detected during the zenith of the sun or moon, but during low tide. A comparison of our moonlight measurements from the intertidal zone (Figure 1a) to expected moonlight in-land (Figure 1b), clearly show that the duration of detectable moonlight levels is shortened by the tide from >12 h to 4-6 h. This is in line with our experimental observation that short natural moonlight (total of 4 h) most effectively entrains the Vigo-2SL strain (Figure 6h).

As the timing of the tides varies along the coastline, the occurrence of a low tide in the middle of the night does rarely coincide with the full moon days (it does for Dinard; Figure 1b and 1c, but not for Vigo and Por Figure 1d and 1e). As a consequence, we can expect from simulations that moonlight availability differs between geographic locations in two major aspects (compare Figures 1c, 1d, and 1e).

First, light in the middle of the night is present on different days of the moonlight cycle. While in Dinard light in the middle of the night occurs at full moon (Figure 1c), in Vigo it occurs several days after full moon (Figure 1d) and in Port-en-Bessin it occurs several days before full moon (Figure 1e). Indirect evidence that these simulated moonlight patterns match the natural situation come from the observed laboratory emergence phenotypes of *C. marinus* strains from the respective (and other) geographic locations. For each strain, the time between artificial moonlight and emergence in the laboratory corresponds well with the time between days with midnight low tides and spring tide days in the field (Kaiser et al., 2011). That means there are strain-specific phase relationships between moonlight perception and adult emergence which match the local tides. These strain-specific phases are genetically determined and can therefore be considered local adaptations (Kaiser et al., 2011). In addition, the observation suggests that the relevant moonlight for setting the phase of the rhythm occurs in the middle of the night, congruent with the observations made in our experiments with moonlight windows.

Second, and concerning the very question of the timing of moonlight during the night, our simulations suggest that the highest moonlight intensity occurs at different times of the night for different geographic locations. While in Dinard it occurs in the middle of the night (Figure 1c), in Vigo it occurs a few hours earlier (Figure 1d), and in Port-en-Bessin it occurs several hours later (Figure 1d). Previous experiments with *C. tsushimensis* (Neumann, 1995) and with the San strain of *C. marinus* (from Santander; Neumann, 1969) have shown that there is a window for moonlight sensitivity only during the night. In addition, the data suggested that this window could have a strain-specific timing. The reason for strain-specific timing of the sensitivity window could be to match the hours of highest moonlight intensity. Alternatively, shifting the timing of moonlight sensitivity could serve to adjust the days during which moonlight is perceived, contributing to adjustment of the phase relationship between light at night and the spring tide days, as described in the paragraph above. In our experiments with different nocturnal moonlight windows (experiments 2a and 2b), the highest moonlight sensitivities (measured as the strongest entrainment of the rhythm) were found to occur in the middle of the night: the Vigo-2NM strain was most sensitive to moonlight just before the middle of the night (Figure 3h) and the Por-1SL strain was most sensitive in the middle of the night (Figure 4k). So in these strains, there is no obvious strain-specificity of the sensitive window. This implies that the sensitivity window does not seem to be adapted to the hours when perceivable moonlight is most intense in a given geographic location; it seems more important to detect if there is light in the middle of the night.

There are two additional lines of evidence in our experiments which suggest that a clear-cut window of moonlight sensitivity is an oversimplistic mechanistic view of moonlight perception, though. First, natural moonlight simulations with naturally low intensity and with modulated light intensities throughout the night and over the lunar cycle resulted in a better entrainment of the rhythm, at least for the lunar rhythm (Experiment 4; Figure 6). The observation suggests that the moonlight perception mechanism is complex in that it also uses information coming from the total light intensity (compare Figure 6f to 6g) and from the gradual change of light intensities (compare Figure 6g and 6h to Figure 5h and 5g). Second, when moonlight is given in windows throughout the night, both strains responded with changing phase relationships between moonlight perception and emergence peak (Figures 3 and 4). There is not a single sensitivity window coupled to a single fix phase of the emergence peak, but for each strain there is a complex relationship between the timing of light during the night and the phase of the rhythm. Even more, fitting the moonlight windows to the patterns of expected light in the natural habitat (Figure 7) suggests that this flexible phase response to light at night compensates for the fact that the hours of moonlight availability shift through the night over the lunar cycle (see Figure 1). It ensures that the timing of emergence stays constant relative to the lunar cycle, no matter when exactly the moonlight is perceived. This is important in making the timing mechanism robust to weather conditions, which may fully obscure moonlight during parts of the lunar cycle. Such compensation is a completely novel finding, and suggests that the complex response to moonlight is an evolved and fine-tuned property of the moonlight perception mechanism.

Notably, for the Vigo-2SL strain the timing of emergence under the moonlight windows corresponds well with field observations (Figure 7c and 7e), while for the Por-1SL strain it does not match at all (Figure 7d and 7f). Thus, for the Por-1SL strain other zeitgebers may be more important than moonlight in adjusting the phase of emergence. This is in line with the observation that the Por-1SL strain does also not seem to be entrained better under the gradual modulation of moonlight (Experiment 4; Figure 6). Other zeitgebers known to entrain the semilunar or lunar rhythm of *C. marinus* are water turbulence (Neumann and Heimbach, 1979) and tidal temperature pulses (Neumann and Heimbach, 1984).

The fact that there is a nocturnal window of moonlight sensitivity also gives an elegant solution to the problem of how to discriminate moonlight and sunlight. If *C. marinus* is only sensitive to moonlight at night, sunlight will not interfere with moonlight detection. Alternatively, or additionally, the discrimination of sunlight and moonlight could rely on differences in wavelengths or light intensities. While we have not tested different wavelengths in our experiments, the fact that the spectral composition of sunlight and moonlight is highly similar (Cohen et al., 2020) argues against a discrimination based on wavelength. We did test light intensities (Experiment 1, Figure 2) and found that moonlight is also well perceived at high intensities corresponding to natural daylight, ruling out that the discrimination is based on the fact that moonlight occurs at low light intensities. Restricting the perception of moonlight to the night remains the most plausible solution in *C. marinus*, although we have shown that the mechanism is more complex than a simple sensitivity window. Notably, in the marine bristle worm *Platynereis dumerilii* a light-receptive cryptochrome (L-cry) adopts different states depending on whether it is excited by bright light (sunlight) or dim light (moonlight). This suggests that in *P. dumerilii* the discrimination of sunlight and moonlight could be based on light intensity (Poehn et al., 2022).

In *C. marinus*, restricting moonlight perception to the night makes entrainment of the lunar rhythm a case of coincidence detection, that is, the moonlight stimulus is only detected when it coincides with a specific time of day (discussed in detail in Kaiser and Neumann, 2021). The fact that moonlight is also perceived in a nocturnal window when it is presented during 4 days of continuous darkness, suggests that a circadian clock is involved in regulating moonlight sensitivity (Neumann, 1995). We may propose that the circadian clock establishes a complex nocturnal light sensitivity window that ultimately coincides with available moonlight during few nights of the lunar cycle. A similar mechanism of circadian dependence for synchronization of lunar rhythms was recently described for corals, where the spawning rhythm in different species was entrained by different gating windows at night (Komoto et al., 2023).

The molecular mechanism by which moonlight sensitivity is regulated over the day in *C. marinus* remains unknown. Interestingly, the shielding pigment of the larval ocelli appears and disappears over the lunar cycle, making the larval ocelli a “radiometer” without a pigment shield at the time of full moon (Fleissner et al., 2008). The same study also assessed the presence of the shielding pigment at different times of day and did not find a circadian regulation, indicating that regulating the shielding pigment is not the mechanism for restricting light sensitivity to the night.

Our experiments suggest that during the time of moonlight sensitivity, a minimum duration of light at night is required. A moonlight window of 2 h was not sufficient to entrain the Por-1SL strain (Experiment 2a, Figure 3). While the Vigo-2NM strain was entrained by a 2-h window of light for four nights, it was not properly entrained by a shifting block of moonlight of 2 h (Experiment 3, Figure 5f). As the same moonlight intensity was applied in the above-mentioned experiments, these results cannot be explained by the high-intensity moonlight treatments used. Rather, it indicates that not only a certain duration of light during the night is required, but also a certain number of successive nights with the same timing of moonlight availability. It matches the observation that a single night of artificial moonlight is not sufficient to entrain the lunar rhythm (Neumann, 1966). There seems to be a summation or integration of light signals on both the daily and the lunar time-scale. Interestingly, in the lunar-rhythmic bristle worm *Platynereis dumerilii*, moonlight duration in the range of 1 to 6 h affects the absorbance spectra of the above-mentioned light sensitive cryptochrome in a duration-dependent manner (Poehn et al., 2022). This suggests that in *Platynereis* the integration of moonlight over the daily time scale may happen at the molecular level.

Finally, our experiments underlined that the second emergence peak observed in the Vigo-2NM strain and other 2NM strains under artificial moonlight (4 nights with continuous high-intensity moonlight) is a laboratory artifact. It is not only absent in the field (Ekrem, Jacobsen, Kokko, Kaiser, in preparation) and under free-run conditions (Neumann, 1966), but also under simulated natural moonlight (Kaiser et al., 2021 supplement and this study). Notably, the natural moonlight simulation presented here is the first laboratory treatment under which both the lunar and the semilunar rhythm of *C. marinus* are precisely entrained without artificial peaks. This lays the basis for QTL mapping for the genetic basis of semilunar (~15 days) vs lunar (~30 days) rhythms, allowing for new avenues of research to finally understand the molecular underpinnings of these enigmatic clocks.

## Supplemental Material

sj-docx-1-jbr-10.1177_07487304241286936 – Supplemental material for How Light at Night Sets the Circalunar Clock in the Marine Midge Clunio marinus Supplemental material, sj-docx-1-jbr-10.1177_07487304241286936 for How Light at Night Sets the Circalunar Clock in the Marine Midge Clunio marinus by Carolina M. Peralta, Eric Feunteun, Julien Guillaudeau, Dušica Briševac and Tobias S. Kaiser in Journal of Biological Rhythms

sj-xlsx-2-jbr-10.1177_07487304241286936 – Supplemental material for How Light at Night Sets the Circalunar Clock in the Marine Midge Clunio marinus Supplemental material, sj-xlsx-2-jbr-10.1177_07487304241286936 for How Light at Night Sets the Circalunar Clock in the Marine Midge Clunio marinus by Carolina M. Peralta, Eric Feunteun, Julien Guillaudeau, Dušica Briševac and Tobias S. Kaiser in Journal of Biological Rhythms

sj-xlsx-3-jbr-10.1177_07487304241286936 – Supplemental material for How Light at Night Sets the Circalunar Clock in the Marine Midge Clunio marinus Supplemental material, sj-xlsx-3-jbr-10.1177_07487304241286936 for How Light at Night Sets the Circalunar Clock in the Marine Midge Clunio marinus by Carolina M. Peralta, Eric Feunteun, Julien Guillaudeau, Dušica Briševac and Tobias S. Kaiser in Journal of Biological Rhythms

sj-xlsx-4-jbr-10.1177_07487304241286936 – Supplemental material for How Light at Night Sets the Circalunar Clock in the Marine Midge Clunio marinus Supplemental material, sj-xlsx-4-jbr-10.1177_07487304241286936 for How Light at Night Sets the Circalunar Clock in the Marine Midge Clunio marinus by Carolina M. Peralta, Eric Feunteun, Julien Guillaudeau, Dušica Briševac and Tobias S. Kaiser in Journal of Biological Rhythms

sj-xlsx-5-jbr-10.1177_07487304241286936 – Supplemental material for How Light at Night Sets the Circalunar Clock in the Marine Midge Clunio marinus Supplemental material, sj-xlsx-5-jbr-10.1177_07487304241286936 for How Light at Night Sets the Circalunar Clock in the Marine Midge Clunio marinus by Carolina M. Peralta, Eric Feunteun, Julien Guillaudeau, Dušica Briševac and Tobias S. Kaiser in Journal of Biological Rhythms
