# Supplementary material for: How Light at Night Sets the Circalunar Clock in the Marine Midge Clunio marinus
Source: J Biol Rhythms. 2024 Nov 6;40(1):91–110. doi: 10.1177/07487304241286936 (PMC11834338; doi:10.1177/07487304241286936)
Supplement: sj-docx-1-jbr-10.1177_07487304241286936 – Supplemental material for How Light at Night Sets the Circalunar Clock in the Marine Midge Clunio marinus [file sj-docx-1-jbr-10.1177_07487304241286936.docx]

## Supplemental Materials

**Supplemental Figures**

Supplemental Figure S1. Geographical origin of *C. marinus* strains and locations of light/water level datasets used in this study.

Supplemental Figure S2. Daylight and moonlight spectra obtained with a radiometer submerged in the intertidal region of Dinard, France.

Supplemental Figure S3. Summed wavelengths of moonlight and daylight over four consecutive lunar months measured  with radiometer submerged in the intertidal region.

Supplemental Figure S4. Most daylight wavelengths are detected across four consecutive months.

Supplemental Figure S5. Light spectra used in experiments 1, 2a, 2b and 3.

Supplemental Figure S6**.** Light intensity modulation in the simulated natural moonlight treatments of experiment 4.

**Supplemental Figure S1. Geographical origin of *C. marinus* strains and locations of light/water level datasets used in this study. (a)** Two populations differing in the period of their lunar rhythm were established and used in this study (Table S1). Vigo-2NM was collected in Vigo, Spain (yellow) and Por-2SL was sampled in Port-en-Bessin, France (dark green). A radiometer was deployed in the intertidal zone of Dinard, France (dark purple) to assess how light intensity and duration is modulated by the tides. Water levels were obtained from the public dataset of ERA5 and relative moonlight data illumination (R package *‘suncalc’*) from the same period of time and from close geographical locations (Dinard, light purple, Por, blue and Vigo, light green).

**
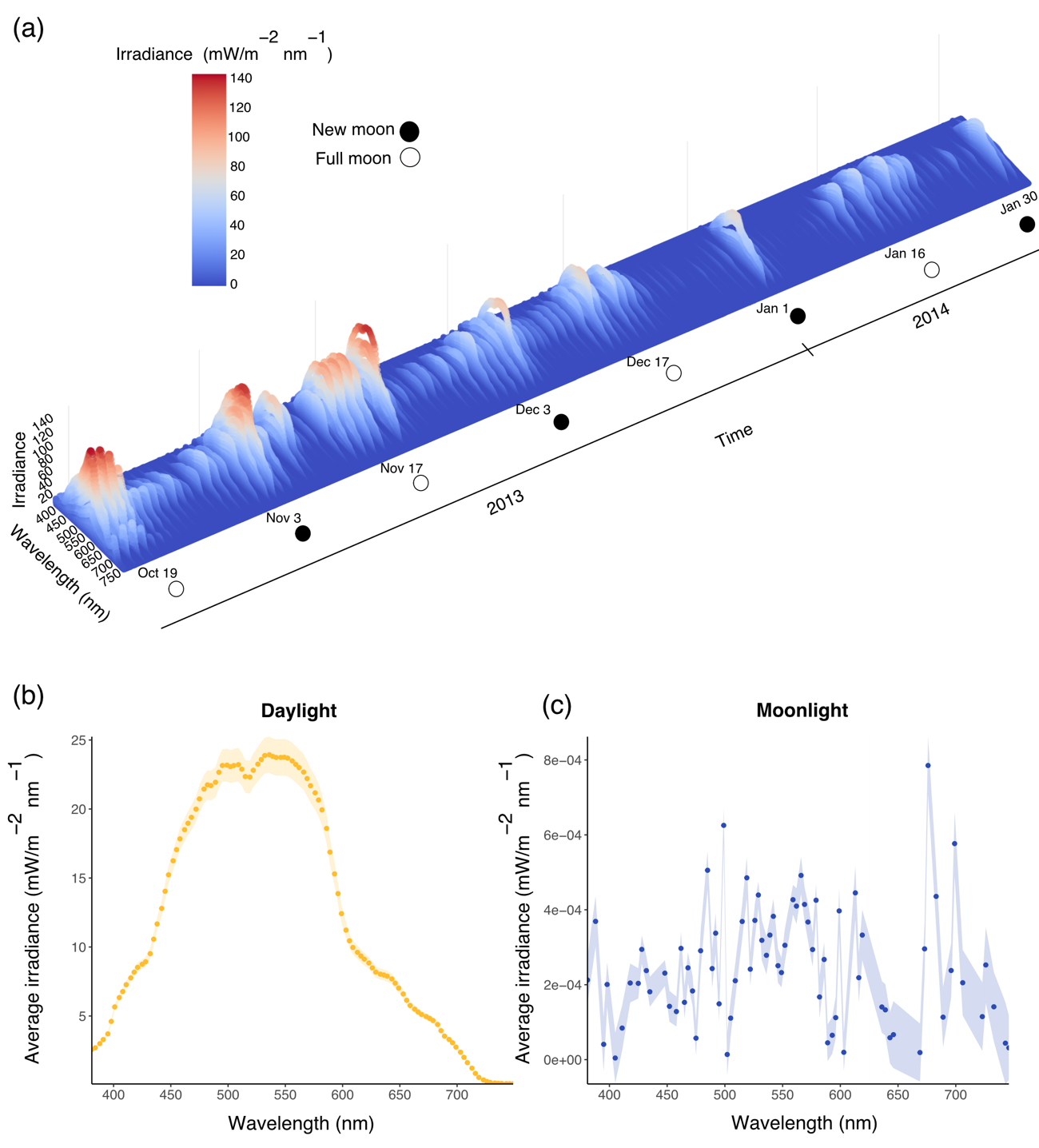
**

**Supplemental Figure S2. Daylight and moonlight spectra obtained with a radiometer submerged in the intertidal region of Dinard, France. (a)** 3D plot of raw light measurements obtained with RAMSES-ACC-VIS hyperspectral radiometer**.** Visible daylight spectrum (380-750 nm) from 17-10-2013 to 01-02-2014 is shown. The highest amplitude of water levels occurs during the spring tides of full moon (white circles) and new moon (black circles), days when irradiance is higher. **(b-c)** Averaged light spectra of two full moon days (2013-11-18 and 2013-12-18) between **(b)** 11h00 and 14h00 to obtain the daylight spectrum and **(c)** 2h00 and 5h00 to obtain the moonlight spectrum. The shading represents the standard error.

**Supplemental Figure S3. Summed wavelengths of moonlight and daylight over four consecutive lunar months measured with radiometer submerged in the intertidal region.** Heatmaps of light intensity with days shown on the x-axis and time of day on the y axis. Five-minute measurements were averaged every 30 min and 192 wavelengths were summed in 100 nm bins. **(a)** Light detected at night between 18h00 and 6h00. Moonlight can be observed between 400 and 699 nm, with the highest intensity (0.032 mW/m^−2^/nm^−1^) detected in 500-599 nm. 0.04 mW/m^−2^/nm^−1^ was employed to establish the upper threshold for all displayed heatmaps, with the minimum allowed value being fixed at 0. **(b)** Light data from (a) normalized within each wavelength bin to the highest value detected in that wavelength bin. The pixels in the 300-399 nm bin and at the beginning of the 700-799 nm and 800-899 nm bins are assumed to be noise. **(c)** Light detected during the day, between 6h00 and 18h00. Light intensity is strongest in days close to spring tides, with the highest value (2780 mW/m^−2^/nm^−1^) in 500-599 nm. Defining the limits of the heatmaps between 0 and 3000 mW/m^−2^/nm^−1^ shows light detected only between 400 and 699 nm. **(d)** Light data from (c) normalized to the highest value detected in each wavelength bin. When normalized, it becomes clear that light is detected in all wavelength bins. White circles below the x axis represent full moon days and black circles show new moon days.

**Supplemental Figure S4. Most daylight wavelengths are detected across four consecutive months.** Heatmaps of five selected wavelengths for each 100 nm bin are shown. Each heatmap is colour-scaled for normalized light intensity (min-max normalization) per wavelength. Days are shown on the x-axis and time of day on the y axis, at a resolution of five-minute measurements. Water levels from the same period of time were obtained from the closest marine station (Saint-Malo) in the publicly available data of maree.info. Highest light intensity at most wavelengths correlates with the timing of low tides. White circles below the x axis represent full moon days and black circles show new moon days.

 **Supplemental Figure S5. Light spectra used in experiments 1, 2a, 2b and 3.** Light spectra was obtained by measuring light with a ILT950 Spectroradiometer. **(a)** Daylight spectrum used for the light-dark (LD) cycle of 16:8. **(b)** Moonlight spectrum used for moonlight entrainment. Moonlight spectrum of the NDUV10B nominal optical density (OD) of 1 does not differ from that of the different neutral density filters used in experiment 1 (data not shown).

**Supplemental Figure S6. Light intensity modulation in the simulated natural moonlight treatments of experiment 4. (a)** Moonlight spectrum of cold white (8000 K) color channel of Mitras Lightbar 2 Actinic 120 (PL-1294, GHL, Germany). The spectrum corresponds to relative light intensity and was extracted from the Profilux Light computer (GHL, Germany) that was used to dim the LED light. **(b-c)** Measurements of light intensity over a full moon night in the simulated natural moonlight programs used in experiment 4. **(b)** “6-hour high intensity” and **(c)** “6-hour low intensity”. The Profilux Light computer calculates light intensities per day and fits the 48- minute shift to a 30-day cycle, causing a jump in light intensity at midnight (b, arrow). The recalculation was set to occur during the day in (c) and thus no shift is observed (compare arrows). Note that light measurements values do not correspond to the ones applied in experiment 4 as the Quantum PAR Radiometer was placed close to the LED light with no filters, for testing purposes.
